# Emerging of Fatal Colitis with Multidrug-Resistant *Candida glabrata* after Small Bowel Transplantation

**DOI:** 10.1155/2021/9995583

**Published:** 2021-09-09

**Authors:** Zahra Zareshahrabadi, Mojtaba Shafiekhani, Hamed Nikoupour, Hasti Nouraei, Hamid Morovati, Kamiar Zomorodian

**Affiliations:** ^1^Department of Parasitology and Mycology, School of Medicine, Shiraz University of Medical Sciences, Shiraz, Iran; ^2^Shiraz Transplant Research Center, Shiraz University of Medical Sciences, Shiraz, Iran; ^3^Basic Sciences in Infectious Diseases Research Center, School of Medicine, Shiraz University of Medical Sciences, Shiraz, Iran

## Abstract

**Background:**

Small bowel transplantation is a potential option for patients with intestinal-failure, and the incidences of infections caused by *Candida* species that are more resistant to antifungal drugs are increasing in these patients. In this manuscript, we reported a case of fatal colitis after small bowel transplantation induces by multidrug-resistant (MDR) *Candida glabrata. Case Presentation*. A 52-year-old man has undergone an extensive small bowel resection with the length of the remaining bowel which was less than 40 cm who became a candidate for transplantation. Four months after transplantation, the patient experienced severe bloody diarrhea with abdominal distension. Ileoscopy and colonoscopy did not show neither pathological change and rejection nor cytomegalovirus (CMV) infection posttransplantation. Abdomen computed tomography showed diffuse moderate small bowel wall thickening. After detection of budding yeast in the stool samples, stool culture was positive for *Candida*, DNA was extracted, and ITS1-5.8s-ITS2 region of the fungal agent was amplified. Sequencing analysis of PCR and antifungal susceptibility testing revealed that this isolate was multidrug-resistant *C. glabrata*. Besides, there was no evidence for other pathogens known to cause infection in various laboratory tests. Immediate antifungal treatments with caspofungin remained unsuccessful, and on the eighteenth day of admission, the patient expires with septic shock.

**Conclusion:**

These findings highlight the challenging management of candidiasis in patients with small bowel transplantation. Infectious diseases due to MDR organisms have emerged as a vital clinical problem in this patient population.

## 1. Background

Infections remain a major cause of morbidity and mortality among solid organ transplant (SOT) recipients. The bacterial followed by viral and fungal infections are the predominant infections which following in SOT [1]. Although fungal infections have remained an encountered challenge among SOT recipients, information on the epidemiology of these infections has been limited mostly to single-center and retrospective studies. The incidence of invasive fungal infection (IFI) among 16,808 SOT patients included in the Transplant-Associated Infection Surveillance Network was estimated at 3.1% [2]. The most common sites of infection are the bloodstream, intra-abdominal, and urinary tract [3]. The incidence of IFI was variable based on the graft type with the highest incidence in small bowel transplant recipients (11.6%) and lowest in kidney transplant recipients (1.3%) [2, 4]. The majority of intra-abdominal fungal infections (40%) are diagnosed in the first month after transplantation, most likely due to the associated disease leading to transplantation, surgical procedure contamination, and loss of the mucosal integrity of intestine during recovery, preservation, and transplantation [5, 6]. In the first 3 months after transplantation, invasive candidiasis as a classic nosocomial infection occurs earlier than other invasive mycoses [7]. Overall, *Candida* spp. are the most common type of fungal infections among SOT recipients except for lung transplant recipients in which *Aspergillus* is more prevalent [2, 8]. Whereas *Candida albicans* has been the most common species isolated from IFIs, there is a steady increase in fungal infections caused by non-*albicans Candida* species [8, 9]. Among the non-*albicans Candida* species, *C. glabrata* is the most common non-*albicans* isolate. The emergence of multidrug-resistant *Candida glabrata*, during prolonged and more broad-spectrum exposure to antifungal agents have created a therapeutic challenge [10]. In this manuscript, we reported fatal severe colitis after isolated small bowel transplantation induces by MDR *C. glabrata*.

## 2. Case Report

### 2.1. History of the Recipient

A 52-year-old man, who developed acute mesenteric ischemia, had undergone an extensive bowel resection. The length of the remaining small bowel was less than 40 cm and accounted as ultrashort bowel syndrome and referred to our center due to evaluation of the possibility of isolated small bowel transplantation (ISTx). He received total parenteral nutrition (TPN) for 18 months and then received a small bowel transplant from donor (14 years brain-dead) with ABO and HLA typing compatible. The patient received methylprednisolone (1 g/day, 4 doses) and thymoglobulin (1.5 mg/kg/day, 4 days) as induction immunosuppressant and then received mycophenolate mofetil, tacrolimus, and prednisolone as maintenance immunosuppressive regimen. Piperacillin-tazobactam (4.5 g q8h for 3 days), vancomycin (1 g q12h for 3 days), and caspofungin (50 mg daily for 2 weeks) started after transplantation as prophylactic antibacterial and antifungal, respectively. He received trimethoprim-sulfamethoxazole and valganciclovir for *Pneumocystis jiroveci* and *Cytomegalovirus* (CMV) prophylaxis, respectively, for the first 6 months after ISTx. The patient was discharged from the hospital after 1 month, and then, outpatient visits were performed in the transplant clinic by transplant surgeons, weekly. Four months after transplantation, the patient experienced severe bloody diarrhea with abdominal distension for three consecutive days. Then, the patient came to the Emergency Rooms (ERs) by ambulance in the hypovolemic shock state with drowsiness. Vital signs on arrival were the temperature of 38°C, blood pressure of 82/57 mmHg, pulse rate of 101/min, and respiratory rate of 24/min (Table 1).

**Figure 1:**
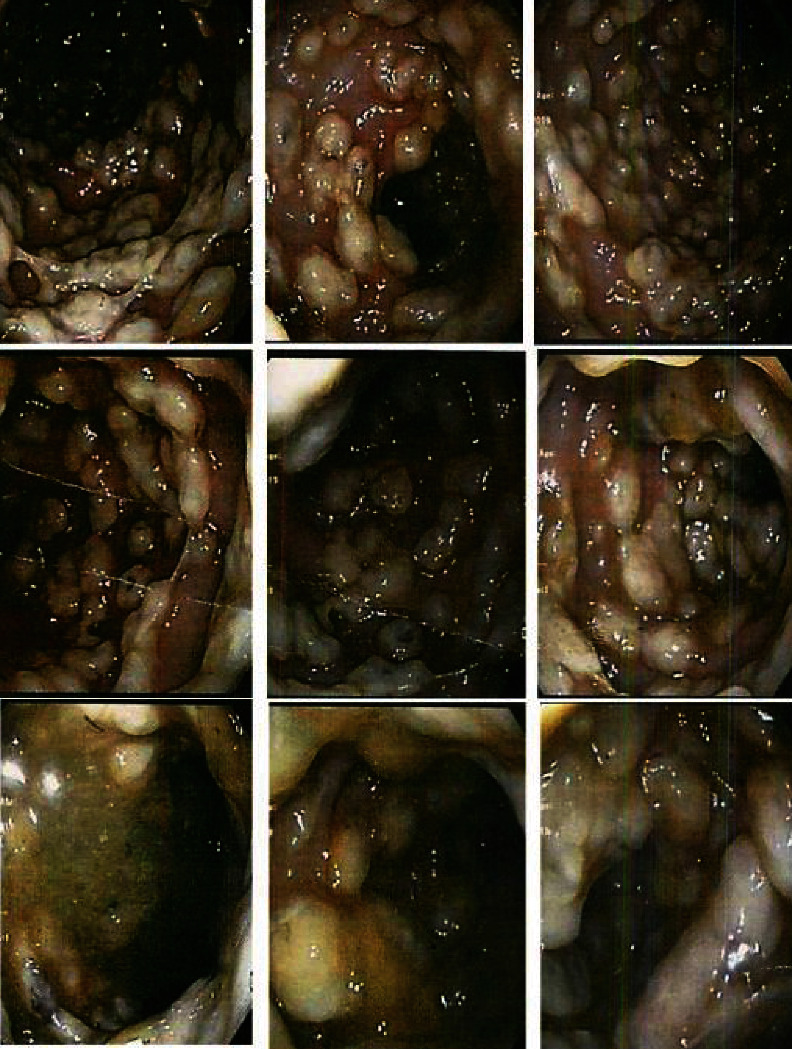
Colonoscopy images show diffuse scattered whitish cobblestone.

The patient was resuscitated with 2 liters of normal saline and transferred to the intensive care unit (ICU) for further evaluation and treatment. On the second day of admission, the patient was intubated, all immunosuppressive was discontinued, inotropes with broad-spectrum antibiotics were started, and sepsis workups were done. The patient underwent diagnostic ileoscopy and colonoscopy. Tissue biopsies did not show neither pathological change and rejection nor CMV organ involvement. Abdomen computed tomography showed diffuse moderate small bowel wall thickening. Stool examination and cultures were analyzed for evaluating infectious causes. Due to colonoscopy features which illustrated diffuse scattered whitish cobblestoning picture (Figure 1) with suspicion of *Clostridium difficile* infection (CDI), detection of fecal toxin A/B by enzyme immunoassay (CDIFF TOX A/B II; TechLab/Wampole, Blacksburg, VA, USA) and culture (CLO agar; bioMérieux, Marcy-l'Etoile, France) were done. All blood, urine, and sputum cultures were negative. Metronidazole plus oral vancomycin were added to his antibiotic regimen to cover suspicious CDI. After detection of budding yeast in the stool samples, caspofungin was started, and more evaluation regarding fungal infection, especially *Candida* spp., was done as below. Stool culture was positive for *C. glabrata* with antifungal susceptibility profiles which are showed in Table 2. On the eleventh day of admission, due to a significant deterioration in the clinical condition and severe abdominal distention, the patient underwent emergency abdominal exploration and total colectomy. Finally, on the eighteenth day of admission, the patient expires with septic shock.

### 2.2. *Candida* Culture and Slide Smear

The stool sample was diluted 1 : 10 with saline, and 100 microliters of dilution was transferred onto a *Candida* CHROMagar (Merck, Germany) and was plated evenly with a sterile swab. After incubation at 37°C for 48 h in ambient air, the *Candida* colonies were counted and classified as *C. glabrata*, according to the color of the colonies. It is notable that a colony count ≥ 10^5^ CFU/ml stool was classified as “*Candida* overgrowth,” according to Krause et al. [11]. In addition, stool sample was examined by light microscopy for the presence of yeasts.

### 2.3. Molecular Evaluation

Molecular evaluation of the *Candida* sp. isolated from the stool sample was performed for the identification of fungal agents. DNA extraction was performed by the boiling lysis method. Single *Candida* colony from a pure fresh Sabouraud dextrose agar (Merck, Germany) plate was picked and inoculated into 200 *μ*l of sterile Milli-Q water and kept for 10 min in a heat block (Rivotek, India) at 100°C. The extracted DNA after incubation at 100°C was kept in a −20°C deep freezer for 10 min and then centrifuged at 10,000 rpm for 5 min. The extracted DNA was stored at -20°C for PCR assay. Amplification of the ITS1-5.8S-ITS2 region was done by universal primers ITS1 (5′-TCC GTA GGT GAA CCT GCG 92G-3′) and ITS4 (5′-TCC TCC GCT TAT TGA TAT GC-3′) at the annealing temperature of 56°C. The amplification was done for 35 cycles of 98°C for 30 s and annealing temperatures of 60°C and 72°C both for 30 s. This was followed by a final extension of 72°C for 5 min. The nucleic acid sequences were compared with the database at the GenBank database using the BLAST sequence search tool. The comparative DNA sequence analysis by nucleotide Basic Local Alignment Search Tool (BLAST) revealed that the amplified sequence was identified as *C. glabrata.* Molecular identification was consistent with culture methods.

### 2.4. Antifungal Susceptibility Testing

The broth microdilution method (CLSI M27-A3/S4) was used for susceptibility testing of our isolate to the following antifungal drugs: fluconazole (FLZ), itraconazole (ITZ), amphotericin B (AMB), caspofungin (CAS) (all from Sigma Chemical Corporation, St. Louis, MO, USA), and voriconazole (VRZ; Pfizer, New York, NY, USA). *C. glabrata* isolate was seeded on the plate containing antifungal drugs, incubated at 37°C for 24 h, and minimum inhibitory concentrations (MICs) were determined by visual examination based on clinical breakpoints or epidemiological cutoff values, which differ across species and the antifungal used. *Candida parapsilosis* (ATCC 22 019) and *C. krusei* (ATCC 6258) were used as references for quality control. Antifungal susceptibility testing found that the *C. glabrata* sample was resistant to FLZ, VRZ, and ITZ, likely resistant to AMB and CAS (Table 2).

## 3. Discussion

Invasive fungal infections are a major problem in SOT recipients. Overall, the most common fungal infection in SOT is candidiasis, followed by aspergillosis and cryptococcosis [4, 12, 13]. Over the last twenty years, intestinal transplantation has been performed for the treatment of patients with intestinal failure and the incidence of fungal infections is higher among patients receiving ISTx than other SOTs because these patients have a central catheter for a long time to receive total parenteral nutrition and broad-spectrum antibiotics and also due to loss of intestinal mucosal integrity during recovery, preservation, and transplantation [12, 14]. In addition, they are susceptible to an intra-abdominal abscesses or intestinal leaks [15]. Invasive fungal infections have been reported in 25.5–59% of the intestinal transplantation recipients [5, 12]. Candidiasis as the most cases of nosocomial infection in intestinal transplantation recipients *Candida* spp. is the most common cause of infection among intestinal transplant patients, which has a role of non-*C. albicans* spp., including *C. glabrata* which is higher than other species of *Candida* genus. To the best of our knowledge, this study is the first case report of severe fetal colitis by *C. glabrata* after ISTx [5, 13]. In general, in patients with small bowel transplantation, in the case of gastrointestinal complication symptoms, especially diarrhea, the first issue that is considered is graft rejection and then CMV infection [5]. Therefore, the occurrence of fungal infections is less considered, which leads to losing the appropriate time to initiate antifungal therapy. Hence, immunosuppressed patients may not show the usual and classic symptoms of fungal infections, so it is necessary to evaluate more carefully in this regard. Arfa et al. revealed that microbial infection was the second common reason for graft failure, after rejection, and they showed in their study that 31% of the patients had a fungal infection, including 64.7% aspergillosis, 17.6% candidiasis, and 17.6% *Pseudallescheria boydii* infection [17]. It is notable that clinical involvement of different species of *Candida* is not similar to each other, so that in our patient who was infected with *C. glabrata* in line with Praneenararat study [16] that reported colitis with *C. tropicalis* agent, dysentery and fever were the early symptoms, while according to the Arfa et al. study [17], colitis with *C. krusei*, fever, and abdominal pain symptoms did not occur, and colitis with diarrhea was the only clinical symptom. In the current case, in addition to the mentioned symptoms, the colonoscopic view was very similar to that of CDI. In SOT transplant recipients, antifungal prophylaxis is usually administered at least for 4 weeks, until anastomosis has entirely healed and resolution of risk factors. Prophylaxis strategies are increasingly used in immunocompromised patients due to the potentially devastating effect of invasive candidiasis in terms of morbidity and mortality. The ideal agent is unclear, but FLZ, CAS, and AMB drugs are logical options [18–20]. Recently, the Clinical Laboratory Standards Institute (CLSI) updated antifungal susceptibility break points for *Candida* spp. [21]. Echinocandin class (ECH) drugs, which inhibit the synthesis of *β*-glucan and disrupt in cell wall integrity, are the first line antifungal therapy against *C. glabrata* infections, as this species has low susceptibility to azole drugs, hereditary [22]. Importantly, resistance to ECH class of antifungal drugs was associated with cross-resistance to azole class in 36% of the cases [14, 23–25] as well as in the current case report study, so that concerns regarding MDR *C. glabrata* significantly increased. The increasing numeral of *C. glabrata* clinical isolates reported showing decreased susceptibility for echinocandins is an emergent concern. According to the previous studies, rates of CAS resistance among *C. glabrata* clinical isolates range from <10% [26] to as high as 62% [27]. Based on the past studies, one possible reason for the increased resistance of *C. glabrata* to the CAS is exposure to low CAS concentrations so that *C. glabrata* is able to colonize and survive in internal parts of the human body, such as the abdomen [28], the peritoneum [29], the gastrointestinal tract [30], or the mucosal surfaces [31], due to long-term penetration of CAS in lower concentrations than those that prevent resistance acquisition. The use of newly developed antifungal drugs that target the 1–3-*β*-D-glucan synthase, such as ibrexafungerp, which has shown potential effectiveness against ECH-resistant *C. glabrata* isolates [32], or rezafungin, which has an extended interval administration due to its improved pharmacodynamics [33], could help to overcome ECH resistance. In our center for patients who undergo a small bowel transplant as long as the patient is NPO, CAS and then FLZ are used for 4 weeks as antifungal prophylaxis. Considering that long-term use of azoles can cause resistance in *Candida* species and, on the other hand, according to the drug resistance pattern reported in this study, it is necessary to reconsider the use of FLZ as part of the prophylaxis regimen.

## 4. Conclusion

In summary, we presented a case of candidiasis with *C. glabrata* agent as the most reported MDR *Candida* spp. in a patient undergoing a small bowel transplant by conventional and molecular analysis. In this study, the triazoles, polyene, and echinocandin classes of antifungal drugs appear to be inactive against *C. glabrata* with high MICs. Further attention is recommended to control fungal pathogens during organ transplantation.

## Figures and Tables

**Table 1:** Patient's clinical and laboratory parameters during hospital stay.

Day of admission	Highest temperature (°C)	WBC (×10^3^/l)	CRP/ESR (mg/l)	PCT (ng/ml)	CMV PCR	TAC (ng/dl)
1	38	13.500	92/108	0.41	Negative	11.29
2	37.7	12.900	92/124	0.41	—	10.11
7	37.5	11.00	64/100	—	—	3.29
14	37.1	14.500	64/82	0.29	Negative	3.51
18	37.6	16.00	64/90	—	—	<2

WBC: white blood cells; CRP: C-reactive protein; ESR: erythrocyte sedimentation rate; PCT: procalcitonin; CMV: cytomegalovirus; TAC: tacrolimus.

**Table 2:** *Candida glabrata* antifungal susceptibility testing results.

Class	Drug	Epidemiological cut-off value	Results from *C. glabrata* isolate	Interpretation
Triazoles	FLZ	≥32 *μ*g/ml	128	Resistant
	ITZ	≥2 *μ*g/ml	64	Resistant
	VRZ	≥0.5 *μ*g/ml	8	Resistant
Polyenes	AMB	≥2 *μ*g/ml	2	Resistant
Echinocandins	CAS	≥0.12 *μ*g/ml	1	Resistant

FLZ: fluconazole; ITZ: itraconazole; VRZ: voriconazole; AMB: amphotericin B; CAS: caspofungin.

## Data Availability

The data used to support the findings of this study were supplied by Shiraz University of Medical Sciences under license and so cannot be made freely available. Requests for access to these data should be made to Kamiar Zomorodian, zomorodian@sums.ac.ir or kzomorodian@gmail.com.
